# The Homeostasis-Enrichment-Plasticity (HEP^®^) Approach for Premature Infants with Developmental Risks: A Pre-Post Feasibility Study

**DOI:** 10.3390/jcm13185374

**Published:** 2024-09-11

**Authors:** Aymen Balikci, Teresa A. May-Benson, Gamze Cagla Sirma, Ayten Kardas, Duygu Demirbas, Ayse Firdevs Aracikul Balikci, Gul Ilbay, Hatice Gulhan Sozen, Isabelle Beaudry-Bellefeuille

**Affiliations:** 1 Sense On, Ltd., Istanbul 34810, Türkiye; 2 TMB Educational Enterprises, LLC., Norristown, PA 19401, USA; tmb@tmbeducation.com; 3 Department of Occupational Therapy, Faculty of Health Sciences, Fenerbahçe University, Istanbul 34758, Türkiye; gamze.dirgen@fbu.edu.tr; 4 Department of Physiology, Faculty of Medicine, Yeditepe University, Istanbul 34755, Türkiye; ayten.kardas@std.yeditepe.edu.tr; 5 Department of Occupational Therapy, Faculty of Health Sciences, İstanbul Sağlık ve Teknoloji University, Istanbul 34275, Türkiye; duygu.demirbas@istun.edu.tr; 6 Department of Special Education, Faculty of Education, Anadolu University, Eskisehir 26210, Türkiye; aysefab@anadolu.edu.tr; 7 Department of Physiology, Faculty of Medicine, Kocaeli University, Kocaeli 41001, Türkiye; gilbay@kocaeli.edu.tr; 8 Department of Child Health and Diseases, Faculty of Medicine, Bahcesehir University, Istanbul 34734, Türkiye; haticegulhan.sozen@med.bau.edu.tr; 9 Clínica de Terapia Ocupacional Pediátrica Beaudry-Bellefeuille, 33007 Oviedo, Spain; ibbergo@gmail.com

**Keywords:** early intervention, environmental enrichment, infant development, premature infant

## Abstract

**Background**: The environmental enrichment (EE) framework has inspired several early intervention (EI) approaches. This study evaluated the feasibility, safety, caregiver acceptance, and satisfaction of implementing the HEP Approach intervention, a novel EI model based on the EE paradigm. Outcome measures for motor development, individual functional goals, sensory functions, caregiver-provided environmental affordances, and motivation for movement were examined. **Methods**: A pre-post-study design examined 18 premature infants (<33 weeks six days gestation) with a corrected age of 4–10 months. A 21-item Likert scale survey assessed the feasibility, safety, acceptability, and satisfaction of implementing the HEP Approach intervention. The Peabody Developmental Motor Scales-2, Test of Sensory Functions in Infants, Affordances in the Home Environment for Motor Development, and Infant Movement Motivation Questionnaire were used for outcomes. The goal attainment scale measured progress toward parent goals. The HEP Approach consisted of 12 one-hour sessions implemented over three months. **Results**: Most participating parents found the HEP Approach intervention feasible, safe, acceptable, and satisfactory. GAS scores demonstrated significant gains with a mean t-score of 67.75 (SD = 2.00). Results found significant improvement (*p* ≤ 0.05) in all outcome measures. **Conclusions**: Results suggest that the HEP Approach intervention is safe, feasible, and acceptable to implement. Outcome measures were meaningful and sensitive in identifying improved motor development, individualized parental goals, sensory functions, caregivers’ use of environmental opportunities, and movement motivation in premature at-risk infants. Results suggest further studies on the HEP Approach are feasible, and highlight the potential of this intervention to inspire and guide future research in this field.

## 1. Introduction [1]

Preterm birth, defined by the World Health Organization (WHO) [1,2,3], refers to births before 37 weeks of gestation. Preterm birth is categorized based on the gestation period at delivery into three subgroups: severe preterm (less than 28 weeks of gestation), extremely preterm (28 to less than 32 weeks of gestation), and moderate to late preterm (32 to less than 37 weeks of gestation). A significant proportion (85%) of premature births take place after 31 weeks of gestation [4]. The most important risk factors for preterm birth are the interplay between the mother’s well-being and the environment, which includes clinical conditions such as maternal age, intrauterine infection, body mass index, inflammation, history of poor obstetric outcomes, short interpregnancy interval, stress, smoking, and a short cervix [2,5,6,7]. The average worldwide occurrence of preterm births is 10.6%, with rates ranging from 8.7% to 13.4% [3,8]. Specifically, the estimated rate of premature birth in Turkey is approximately 11.9% [9], slightly higher than the worldwide average, thus making support for preterm infants a high priority. 

Many factors occurring before, during, and after birth might impact the neurodevelopment of preterm newborn, increasing their susceptibility to difficulties in sensory, motor, cognitive, and behavioral areas [4,10,11,12]. Although the majority of children born prematurely before 34 weeks of gestation may not face significant impairments, a larger proportion of these preterm children, compared to those delivered at full-term, face neurodevelopmental challenges such as sensory-motor and attention deficits, learning difficulties, autism-like symptoms, developmental delay, and cerebral palsy [10,13,14]. While most research has focused on studying extremely premature infants, there is a growing recognition that infants born between 34 and 37 weeks of gestation also face higher rates of mortality, morbidity, and neurodevelopmental difficulties [4,10,11,13,15].

Early intervention can mitigate the impact of social and biological risks and yield advantages over their lifespan for premature infants and their families [14,16,17]. Early intervention refers to programs implemented immediately after a child’s birth and lasting until age 3 [18]. These programs provide a range of services to children who are at risk as well as their families [16]. The goal of early intervention is to promote the health and well-being of the child, help them develop essential abilities, prevent delays in their development, reduce any existing disabilities, prevent loss of functionality, strengthen the bond between the caregivers and child, help the family adjust to having a child with special needs, and promote effective parenting and overall family functioning [14,16,19].

In the last decade, numerous early intervention programs rooted in family-based ecological models have addressed the developmental risks faced by premature infants [14,16,20,21,22,23,24,25,26,27,28]. These recent intervention approaches, in contrast to conventional methods that focus on neurologic and musculoskeletal maturation, consider the impact of the environment on the child’s development [20,29,30,31,32,33]. Furthermore, the environmental enrichment (EE) paradigm has influenced multiple novel early intervention approaches [18,20,23,34,35,36] based on significant favorable structural and functional outcomes in animal experimental research [37,38,39,40,41,42]. 

Environmental enrichment (EE) research involves exposing laboratory animals to complex surroundings designed to promote their sensory, social, motor, and cognitive abilities in contrast to the ordinary living conditions typically found in animal laboratories [37,40,42,43]. Experimental EE paradigms involve spacious spaces that contain a variety of tunnels, ladders, shelters, and toys with different shapes, sizes, and textures [38,39,40,43]. These setups offer sensory stimulation and opportunities for laboratory animals to engage in their natural behaviors in supportive environments. This environment aims to enhance the functioning of the nervous system and foster the development of problem-solving skills. Moreover, the objects are periodically rearranged and substituted to improve the animals’ exploration, attention, and curiosity. Animals are raised in big groups to experience stimulating smells, sights, and sounds of other animals, which promotes cooperation, fighting, and playful behaviors [40,42]. 

Although the enrichment conditions may vary in EE studies, they commonly share fundamental features and have consistently demonstrated beneficial outcomes [40]. The essential features of EE can be described as follows: providing sensory-motor experiences, incorporating spatial features of the environment, introducing environmental and item novelty, problem-solving, and providing challenges [43,44,45,46]. Research suggests that EE supports the development of both brain structure and behavior, so early intervention programs designed to support premature infants at risk of developmental challenges must align with the fundamental principles of EE [39,45]. 

The Homeostasis-Enrichment-Plasticity (HEP) Approach for early intervention was developed to incorporate the fundamental principles of EE within ecological theories of human development, emphasizing the essential role of homeostasis in the developmental process [45,46]. The HEP Approach is designed to be used in physical and occupational therapy by pediatric therapists with a solid knowledge base in early child development. This study aimed to examine the feasibility, safety, caregiver acceptability, and satisfaction of implementation of the HEP Approach, and the sensitivity of meaningful outcome measures for motor development, individualized functional goals, sensory functions, the provision of environmental affordances by the caregivers, and movement motivation of premature infants at risk for developmental delays.

## 2. Materials and Methods

### 2.1. Study Design and Procedures

This is a pre-post feasibility study. The study has been approved by the Fenerbahce University Clinical Research Ethics Committee (012024fbu). Caregivers provided informed consent. The study was conducted at Fenerbahçe University Istanbul-Turkey. 

The study consisted of four phases. Phase one included participant recruitment and introducing the families to the project. Phase two included an initial comprehensive assessment, goal setting, and data collection. Phase three consisted of 12 weeks of intervention (see Table 1 and Table S2 for details on the intervention). Phase four consisted of a post-testing assessment and data analysis. Participants were recruited as described below. Data were collected pre- and post-intervention via comprehensive assessment, goal attainment, and a feasibility questionnaire as described in the measures sections. Fidelity to the intervention was assured through the mentoring of the treating therapists and a review of the intervention sessions with the first author. 

### 2.2. Participants and Recruitment 

Non-probability convenience sampling was utilized. Premature infants who matched the specified inclusion criteria were referred by neonatologists working in three hospitals in Istanbul. Inclusion criteria were as follows: (a) corrected age between 4 and 10 months; (b) birth at or before 33 weeks six days of gestation; (c) absence of systemic disease or congenital disabilities; and (d) agreement by the family to engage in the study process regularly. Exclusion criteria encompassed the presence of the following conditions: (a) prior occurrence of intraventricular hemorrhage of grades iii or iv; (b) significant impairment of vision or hearing; (c) history of febrile convulsion; (d) medical conditions that hinder active participation in the study such as reliance on oxygen; and (e) prior involvement in other experimental rehabilitation studies. Upon verification of the eligibility criteria, informed consent was obtained.

**Figure 1:**
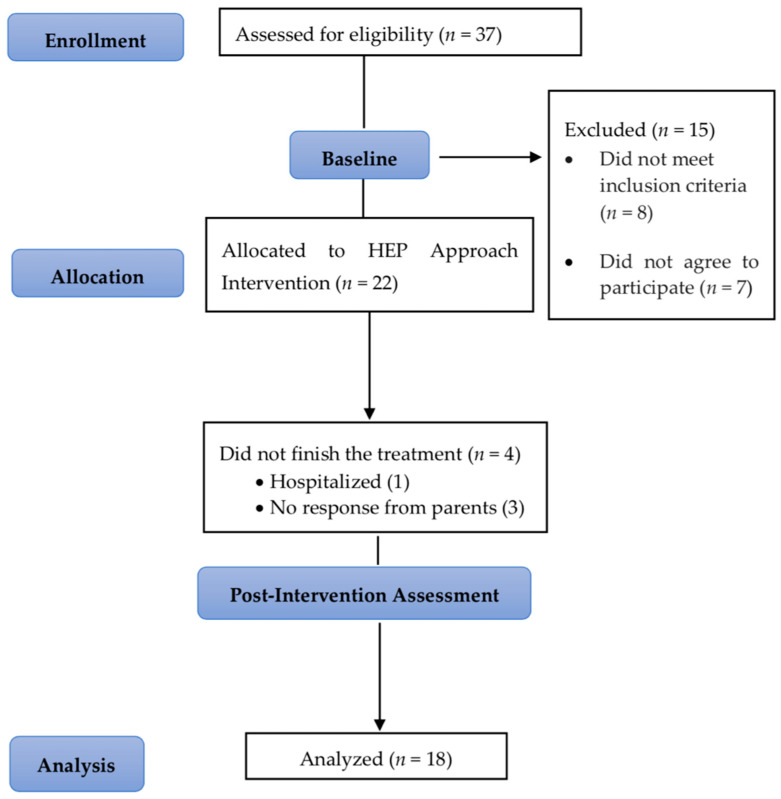
Flowchart of the recruitment and intervention process of the participants.

A total of 37 preterm infants were referred to the study. Eight participants failed to meet the inclusion criteria, and seven families did not engage in the study. A total of 22 infants participated. Four infants were eliminated after the start of the study due to one requiring hospitalization and the inability to communicate further with the families of the others. Ultimately, 18 infants completed the intervention. Figure 1 displays the flowchart illustrating the involvement of infants in the study.

Two therapists, a physiotherapist and an occupational therapist specializing in pediatrics with over five years of experience and completing an advanced Master of Science degree in their respective fields, participated as outcome evaluators. These therapists received training on administering the outcome measure assessments from the first author, a PhD physiotherapist with over 15 years of pediatric experience. These therapists only administered the outcome measures and did not participate in the intervention phase of the study. The same examiner administered the infants’ evaluations before and after the intervention. The first author completed outcome measure scoring.

The HEP Approach intervention was provided by a physiotherapist and an occupational therapist, each with over five years of pediatric experience and participation in advanced Master’s programs. The therapists were blind to all of the testing results. The first author, the developer of the HEP Approach, provided training and supervision on the HEP Approach.

### 2.3. Feasibility Measure

The researchers developed a 21-item Likert scale survey to assess the feasibility, safety, acceptability, and satisfaction of intervention implementation. The survey was comprised of items categorized under four primary headings: feasibility (items 8, 12, and 14), safety (item 21), acceptability (items 6,7, 9, 10, 11, and 13), and satisfaction (items 1 to 5, 15, and items 17 to 20). The Likert survey consisted of five response options: Strongly Agree, Agree, Neither Agree Nor Disagree, Disagree, and Strongly Disagree. For this study, responses indicating “Strongly Agree” and “Agree” were classified as agreement, while all other responses were categorized as disagreement. Results were analyzed in terms of percentages (see Table S1).

The treating therapists also completed a separate five-question survey at the end of the study. The questions asked about safety, feasibility of implementing the intervention, and acceptability of the study and intervention procedures.

### 2.4. Outcome Measures

The caregivers completed a demographic survey to collect data on the health of the infant and mother and information on gestational and postnatal factors. Questions examined the infant’s corrected enrollment age, gender, birth weight, gestational age, maternal age, family income, and the caregivers’ educational level.

Five outcome measures were identified to examine the assessments’ ability to detect change in areas of gross and fine motor skills, sensory processing, parental functional goals, child motivation to move, and the presence of facilitating environmental affordances in the child’s home environment.

#### 2.4.1. Goal Attainment Scaling (GAS)

Meaningful functional, individualized, caregiver-established goals were measured using goal attainment scales (GAS). GAS is the most recommended goal-setting methodology for measuring change during and after intervention in clinical and research applications. Research shows that GAS is a reliable tool for children with developmental challenges. GAS provides subjective information about the infant’s needs. It is a method of measuring the extent to which the child’s individual goals, set at the start of the intervention, are achieved. A 5-point scale (−2 to +2) is used for scaling goals. The predicted level of performance is scored as a 0, with −1 indicating somewhat less than the expected outcome, −2 much less than the expected outcome, +1 somewhat more than the expected outcome, and +2 much more than the expected outcome [47,48,49]. This study used the goal scaling and scoring methodology advocated by Kiresuk et al. (2014) [49].

#### 2.4.2. The Peabody Developmental Motor Scales-2 (PDMS-2)-Turkish Adaptation

The Peabody Developmental Motor Scales-second edition (PDMS-2) evaluates motor development. It consists of six subtests that examine interconnected skills in early motor development and is designed to assess children’s gross and fine motor abilities from infancy to 5 years old. A total motor quotient (TMQ) is calculated from the test’s subscores. The TMQ is considered the most representative assessment of general motor skills. The gross motor quotient (GMQ) and the fine motor quotient (FMQ) scores are calculated from specific unique subscores. The GMQ examines locomotion, postural control while stationary, response to environmental changes, and successful grasp and release of objects. The FMQ examines manual dexterity, object manipulation, hand grasp, and drawing. The test takes approximately 45 to 60 min to administer, although it may be shorter for infants [50,51]. The PDMS-2 is a reliable and valid tool for evaluating the motor development of premature infants [51,52]. A Turkish cultural adaptation of the measure was used for test administration, but the standard test norms were used because Turkish norms were unavailable.

#### 2.4.3. Test of Sensory Functions in Infants (TSFI)-Turkish Adaptation

The TSFI is a standardized therapist-administered assessment of sensory functioning for infants between the ages of 4 and 18 months. The TSFI is routinely used with infants with regulatory disorders, developmental delays, and infants at risk of developing sensory integration disorder. The test comprises 24 items divided into five sensory processing and reactivity subtests. The subtests include reactivity to deep touch pressure, adaptive motor functions, visual-tactile integration, ocular–motor control, and reactivity to vestibular stimulation. Results are reported using standard z-scores with scores above −1 SD considered “Normal”, scores between −1 SD and −2 SD being “At risk”, and scores below −2 SD as “Deficient” [53,54]. A Turkish cultural adaptation was used for test administration, and Turkish normative data were used for scoring [54].

#### 2.4.4. Affordances in the Home Environment for Motor Development (AHEMD-IS)-Turkish Adaptation

AHEMD-IS is a reliable and valid measure used to evaluate the quantity and quality of engagement opportunities provided by the family and the home environment [55,56]. The questionnaire comprises four dimensions: physical space, variety of stimulation, fine motor toys, and gross motor toys. The raw score is calculated by summing together the scores of the four dimensions. The Turkish adaptation of the AHEMD-IS assessment has been shown to possess both validity and reliability whether used with children born prematurely or at full-term [56]. A Turkish cultural adaptation was used for test administration, and Turkish normative data were used for scoring.

#### 2.4.5. Infant Movement Motivation Questionnaire (IMMQ)-Turkish Adaptation

The Infant Movement and Motivation Questionnaire (IMMQ) assesses the level of motivation in infants aged 0 to 12 months to engage in physical movement. The questionnaire consists of 27 items intended to be completed by parents or caregivers. It comprises four factors: activity, exploration, motivation, and adaptability. The IMMQ has good internal consistency with a Cronbach’s alpha of 0.89. The test–retest reliability of the IMMQ is strong, with an ICC coefficient of 0.92 and a 95% confidence interval ranging from 0.83 to 0.96 [57]. The Turkish adaptation of the IMMQ was completed by Altunalan E. (2020), and it has been established as a reliable and valid instrument for usage by the Turkish population [58]. A Turkish cultural adaptation was used for test administration, and Turkish normative data were used for scoring.

### 2.5. Intervention

The HEP Approach intervention consisted of 12 one-hour sessions conducted over three months. Clinic-based intervention was completed in an 8 × 8 foot room with a parent and therapist present with the child. The two therapists provided clinic-based parental/caregiver coaching weekly in this setting. The room was equipped with a variety of standard sensory integration and gross and fine motor therapeutic materials such as gym balls, foam wedges, barrels, and rolls. All intervention sessions were videorecorded. Fidelity to the intervention approach was assured through weekly reviews of the intervention videos and input provided by the first author to the therapists regarding their adherence to the main principles of the HEP Approach. Parents were encouraged to implement strategies presented in the clinic sessions within the home environment. Families provided videos to their therapists every week using WhatsApp. Caregivers then received valuable comments and encouragement from the therapist.

The HEP Approach intervention and clinical reasoning process follow a systematic order based on the data-driven decision-making (DDDM) model [59]. This process is described in Table 1. There are 11 phases to the HEP Approach process. Phase 1 involves referral to the program. Phase 2 includes a meeting with the family to introduce them to the HEP Approach. Phase 3 is a comprehensive assessment of the child and family systems. Phase 4 is the identification of family and child strengths and challenges based on the assessment. Phase 5 involves formulating hypotheses about how underlying factors or systems impact the child’s challenge areas. Phases 6 and 7 include collaborative goal setting and outcome measure identification with the family. Phase 8 is intervention planning. Phase 9 implements the intervention through an individualized process that generally involves four steps that prioritize different areas of need (e.g., self-regulation and homeostasis of the child, adaptation of the physical and social home environment to support success, generalizing and diversifying interactions with the environment, and supporting family independence and autonomy in creating supportive environments for the child). Phase 10 involves family monitoring and follow-up and, finally, Phase 11 involves the assessment of goals attainment and progress.

The HEP Approach utilizes key principles of enriched environment models and brain plasticity, which are commonly employed in experimental animal research. It uses these principles within the framework of ecological theories of human development and highlights the crucial role of homeostasis in the development process [45,46]. The therapists and family collaborated during the intervention process to offer permanent, ongoing, personalized environmental stimulation to promote the child’s active exploration and participation. A crucial component of the HEP Approach is enhancing parenting self-efficacy to create enriched environmental conditions in the home and on an ongoing basis across environments. The HEP Approach consists of 10 fundamental intervention principles (defined by Balikci, 2022; see Table S2 for details) mainly based on EE studies [45]. These principles include the maintenance of physiological homeostasis, provision of sensory experiences, safety, environmental and object novelty, changing spatial features of the environment, just-right challenge, enjoyment, social opportunities, continuous engagement with the environment, and active engagement in and exploration of the environment. The HEP Approach integrates these principles with the fundamental tenets of dynamic systems theory, Gibson’s ecological theory of perception, the theory of neuronal group selection, sensory integration, and the person-environment-occupation model [45,46]. Table S2—HEP Approach Intervention Guide presents definitions of the ten fundamental characteristics of the HEP Approach intervention and offers examples of the clinical application of the principles.

### 2.6. Analysis

Study data were analyzed using the IBM SPSS Statistics for Windows, Version 22.0 (SPSS INC., Chicago, IL, USA) statistical software. Descriptive and demographic data were reported using frequency and percentage analyses. Outcome measure scores were examined using mean and standard deviation statistics. Skewness and kurtosis were examined for normality of the research variables. Variables with kurtosis and skewness values between +1.5 and −1.5 [60] and between +2.0 and −2.0 [61] were deemed to follow a normal distribution. The variables in this study exhibited a normal distribution. Therefore, data were analyzed using parametric approaches. A paired *t*-test was employed to compare repeated measures for the dependent groups. A *p*-value less than 0.05 indicated statistical significance.

Five GAS goals were written for each participant. For these parent-identified GAS goals, the mean score for each participant was calculated and converted to a t-score [62]. A mean GAS t-score was then calculated for the group. A mean group t-score of 50 or higher indicated achievement or performance that exceeded the expected goal performance.

## 3. Results

### 3.1. Demographics

Table 2 summarizes the characteristics of the 18 families and infants who completed the HEP Approach intervention. The mean corrected age was 26.06 (SD = 7.13) weeks, mean birth weight was 1.48 kg (SD = 0.34), mean gestational age was 30.67 (SD = 2.74), and mean maternal age was 30.44 (SD = 2.47). Of the eighteen newborns that participated, eight were female; ten were male. A total of 16.7% of parents had family incomes ranging from 1 to 2 times the minimum wage, while 44.4% had incomes ranging from 3 to 4 times the minimum wage. The remaining 38.9% of parents had family incomes exceeding five times the minimum wage. Education levels among the mothers were high school (*n* = 4, 22.2%) to bachelor’s degree (*n* = 11, 61.1%), and MSc or PhD (*n* = 3, 16.7%) for mothers. Among the 18 fathers, 3 (16.7%) had a high school degree, 15 (83.3%) had a bachelor’s degree, and none had a master’s or doctoral degree.

### 3.2. Feasibility, Safety, Satisfaction, and Acceptability of the Intervention

Feasibility of the intervention was assessed using items 8, 12, and 14 on the survey. According to 72.2% of parents, the HEP Approach intervention was logistically convenient for them. In addition, 95.5% found it easy to attend in-person sessions every week, and 100% found it easy to implement HEP Approach activities into their daily routines.

Item 21 of the survey assessed the intervention’s safety. Parents indicated that they considered the HEP Approach activities safe for their infants (100%).

The survey assessed caregiver satisfaction of the intervention procedures using items 1–5, 15, and 17–20. Parents indicated that they received sufficient information regarding the intervention process and the study, and they expressed complete satisfaction with the HEP Approach intervention given to their infant (100%). They stated that they would wholeheartedly endorse the HEP Approach intervention to other caregivers of premature infants, with a 100% recommendation rate. The parents reported that the therapist successfully formed a pleasant relationship with their infant and effectively communicated with them. As a result of the HEP Approach intervention, their infants demonstrated a noticeable improvement in calmness (100%). Parents also reported that the HEP Approach intervention positively impacted their baby’s movement development, with a satisfaction rate of 94.5%. In addition, they noted that the HEP Approach intervention had a beneficial impact on their baby’s cognitive development including attention, problem-solving, and object permanence. They also reported that the intervention improved their child’s exploratory abilities and greatly affected their family dynamic, with a 100% satisfaction rate.

The acceptability of the intervention procedures among caregivers was assessed using items 6, 7, 9, 10, 11, and 13 on the survey. Parents indicated that the infants experienced happiness and enjoyment throughout the therapy sessions. They found the HEP Approach intervention appropriate and beneficial for their infants, with a 100% acceptance rate. A total of 88.9% of parents expressed interest in participating in a future study on the HEP Approach intervention. Additionally, they reported that the duration of the sessions was acceptable, and they found it highly beneficial to have the therapist monitor how they were doing at home through video sharing. Furthermore, caregivers found it convenient to attend in-person sessions weekly, with a 100% attendance rate.

Both therapists implementing the HEP Approach indicated that the time spent each week on intervention preparation and providing family support outside of therapy sessions was practical and easily manageable (100%) and found the procedure of supporting the family through shared activity videos to be satisfactory (100%). They reported satisfaction with the level of supervision they received for the intervention (100%). Treating therapists reported that the HEP Approach intervention activities and procedures appeared safe, with no documented adverse outcomes (100%). In addition, the therapists stated that they found the HEP Approach procedures to be acceptable and in line with their theoretical and practical knowledge (100%).

### 3.3. Goal Attainment Scaling (GAS) Outcomes

Goal attainment scales generated before intervention were rated by the parents post-intervention. Table 3 provides a sample GAS scale. Meaningful functional goals about motor development (i.e., sitting, rolling, creeping), manual exploration (i.e., handling, shaking), self-regulation (i.e., calming down quickly when crying), sensory processing (i.e., tolerating sounds and touch), and relationship or communication (i.e., engaging in a cycle of communication with adults or responding to their expressions) were most commonly set by parents. GAS scores demonstrated significant improvements, with a group mean GAS score of +1.14. The mean GAS t-score was 67.75 (Min = 65.08, Max = 71.11, SD = 2.009, N = 18), indicating somewhat more than the expected gains.

### 3.4. The Peabody Developmental Motor Scales-2 (PDMS-2)

The PDMS-2 was found to be sensitive to changes in motor performance. Significant gains were found in the standard scores on the PDMS-2 subtests (reflexes, stationary, locomotion, grasping, and visual-motor integration) (*p* < 0.05). The GMQ, FMQ, and TMQ scores also displayed statistically significant improvement (*p* < 0.05) following the HEP Approach intervention (See Table 4).

### 3.5. Test of Sensory Functions in Infants (TSFI)

The TSFI was found to be sensitive to changes in sensory processing. All subtests (reactivity to tactile deep pressure, adaptive motor function, visual tactile integration, ocular motor control, reactivity to vestibular stimulation) and total scores demonstrated significant improvement (*p* < 0.05) after the HEP Approach intervention (See Table 4).

### 3.6. Affordances in the Home Environment for Motor Development (AHEMD-IS)

The AHEMD-IS was sensitive to changes in affordances present in the home environment. Except for the AHEMD-IS “Physical Space” subsection, all subsections (variety of stimulation, fine-motor toys, and gross-motor toys) showed significant improvement (*p* < 0.05) (see Table 4).

### 3.7. Infant Movement Motivation Questionnaire (IMMQ)

THE IMMQ is sensitive to changes in the child’s motivation to move and engage in the environment. The IMMQ’s subsections of activity, exploration, motivation, and adaptability as well as the total scores showed a statistically significant improvement following the HEP Approach intervention (*p* < 0.05) (see Table 4).

## 4. Discussion

This study aimed to investigate the feasibility of utilizing the HEP Approach with high-risk premature babies and examined the acceptance and satisfaction of families and staff regarding this approach. All families (100%) reported the HEP Approach intervention as acceptable, beneficial, convenient to use in everyday routines, and safe for their infants. Moreover, they reported that the outcomes of the HEP Approach were satisfactory, and all participants endorsed this therapy paradigm for parents of preterm infants. These results are similar to the perspectives of families who previously engaged in early intervention programs influenced by EE [20,34,36,63]. Furthermore, outcomes were comparable to previous case reports of children with cerebral palsy [45] and twin anemia polycythemia sequence (TAPS) [46] who participated in the HEP Approach intervention.

According to the study conducted by Morgan et al. (2016), parents of infants who received the “GAME” (Goals-Activity-Motor Enrichment) intervention for 16 weeks reported a high level of satisfaction with their infant’s accomplishments [20]. Another study found that the “Specific Task-Environment-Participation” (STEP) protocol, another EE-based EI program, had good applicability and was easy to carry out. Parents recommended this treatment model and were satisfied with the positive changes in their infants after the intervention [36]. The families’ favorable response to EE-based EI approaches appeared to be related to their active engagement in the intervention. In the current study, caregivers reported high levels of satisfaction with the support provided with the HEP Approach. Support areas included caregiver empathetic inquiry, mindfulness, self-regulation, capacity building, collaborative exploration, and reflection. Furthermore, the research demonstrated that involving caregivers in the therapeutic process enhanced participation in goal setting, decision-making, the identification of more favorable and stimulating conditions for development, and collaboration with healthcare experts, corroborating our findings [36,64,65,66].

Achieving an outcome that exceeded expectations after implementing the HEP Approach intervention resulted in meaningful, functional changes in caregiver-established goals assessed by GAS. These results are consistent with prior research, which found that early intervention programs based on EE significantly affected the attainment of parental objectives [20,23,67]. Morgan et al. (2015) found that the EE-based GAME intervention improved parent-established GAS goals [67]. Another GAME intervention study utilized the Canadian Occupational Performance Measure (COPM) to determine parents’ principal goals regarding their infant’s development and found significant improvements in COPM performance after 16 weeks [20]. A study by Apaydin et al. (2023) produced positive outcomes in satisfaction and performance on the COPM for preterm infants using the SAFE (Sensory Strategies, Activity-Based Motor Training, Family Collaboration, and Environmental Enrichment) program, which is also grounded in EE [23].

The outcome measures used in this study provided meaningful and sensitive results. The HEP Approach intervention resulted in a statistically significant improvement in all measures. Significant improvements in infant gross motor, fine motor, and total motor development were found in the subtest standard scores and quotient scores of the PDMS-2. The results of this study are consistent with previous studies that showed positive effects of EE-based interventions on the motor development of infants [23,36,37,67,68,69]. A recent study found that the EE-based STEP protocol improved the performance of motor skills in infants at risk of developmental delays compared to traditional physical therapy intervention [36]. Furthermore, Apaydin et al. (2023) found that the SAFE approach yielded favorable outcomes for preterm infant motor development [23]. A study using the EE-based CareToy training program for four weeks, utilizing goal-oriented activities to motivate premature infants to accomplish specific tasks, significantly improved their motor performance and visual development [22]. In addition, Morgan et al. (2016) showed that the GAME intervention model was more successful than standard care in enhancing the motor performance of infants [20]. Finally, another approach rooted in the EE paradigm, SPEEDI (Supporting Play, Exploration, and Early Development Intervention), also demonstrated improvements in motor development [27,34]. Thus, the results of this study are consistent with previous research on EE-based interventions.

Although existing research has investigated the impact of EE on motor development, few studies have explored the effect of EE-based interventions on the sensory functioning of infants. This study and previous case reports and studies by the first author and colleagues [45,46] found that the HEP Approach significantly improved the sensory functions of infants with various developmental challenges. Apaydin et al. (2023) conducted a recent study that corroborated these findings [23]. They found that the SAFE intervention positively affected the sensory processing of premature infants, as measured by the TSFI. Zhang et al. (2022) demonstrated a positive impact on the sensory processing abilities of preschool children through a specific functional training program to enhance motor development [16]. This study emphasized the importance of motor skills in developing sensory processing abilities. Moreover, in the current study, the child’s increased involvement in the active exploration of objects and the environment and the sensory experiences resulting from the development of motor abilities may explain the enhancement of sensory functions in the current study [70,71,72].

The AHEMD-IS found significant and favorable improvements in the infants’ active exploration and motor development in their home environment. Families were guided by their therapist to create these environmental opportunities according to the ten fundamental principles of the HEP Approach. Thus, families were guided to provide their babies with a safe, comprehensive, diverse, sensory-rich, and meaningful social and physical environment. Supporting literature validates these findings [73,74]. Zorlular et al. (2024) suggest that organizing the home environment and establishing favorable settings during the early stages of life can positively impact the development of premature infants [74]. Furthermore, Apaydın et al. (2023) provided evidence that the SAFE approach significantly improved the ability of the home environment to promote motor development and sensory processing skills, as assessed by AHEMD-IS [23]. Another study by Morgan et al. (2016) found that the average scores in the GAME intervention group demonstrated a proportional increase in the AHEMD-IS in relation to the maximum achievable score on the measure during the period of the study [20]. They determined that although the change was not statistically significant, the findings suggest that parents in the GAME intervention group were more likely to provide a broader range of experiences to address motor performance as their child’s skills improved. The HEP Approach is unique because it utilizes reflective questions to guide families in establishing appropriate environmental conditions for their infants. This reflective component of the HEP Approach aims to enhance the family’s capacity to participate as an active partner in the intervention process, leading to their increased capacity to make informed decisions on their own and not simply apply the recommendations made by the therapist. Consequently, the HEP Approach enables families to address ongoing demands and promote their infants’ active exploration and engagement outside direct intervention.

Recent research also suggests that motivation is crucial in developing motor skills and behaviors. It is recommended that the variability in newborns’ urge to move impacts when infants reach their motor milestones [57,75]. The results of this study and those found in the literature are comparable. Development of sensory-motor skills occurred in concert with an increase in movement motivation. Recent research further corroborates these findings, demonstrating that changes in one developmental domain impact other developmental domains [75,76].

This study had some limitations. First, it should be acknowledged that while control groups are not expected in feasibility studies, in this particular case, there was indeed no control group present. The sample size of 18 children was small, which reduces the generalizability of findings, but it has adequate power for an initial feasibility study. Future randomized controlled trials should include a larger sample. This sample was relatively homogeneous, and examination of other diverse challenges would also be helpful in the future. Furthermore, the impact of the HEP Approach intervention on social-emotional and cognitive development domains and parental health was not assessed. Studies that incorporate a control group and assess additional aspects of development as well as the impact of the intervention on the family will provide significant contributions in this regard. The next phase of examination of the HEP Approach would be to conduct a randomized controlled trial.

## 5. Conclusions

The results of this study suggest that the HEP Approach intervention is safe, feasible, and acceptable to implement clinically and for most parents of preterm infants to participate in. Almost all participating parents reported that implementing the HEP Approach intervention strategies at home was easy and favorable for their infants. Thus, the HEP Approach intervention aligns well with family-centered EI practice and incorporates parental empowerment, a crucial element.

This study demonstrated the feasibility of carrying out the ten fundamental principles of the HEP Approach, which is grounded in EE studies, with premature infants between the ages of 4 and 10 months at risk for developmental delays. The outcome measures used were meaningful and sensitive in identifying improved motor development, individualized parental goals, sensory functions, the caregivers’ use of environmental opportunities, and the movement motivation of premature infants who are at risk. These results suggest that further studies on the HEP Approach are feasible. Due to the absence of experimental controls in this research, it is impossible to conclude the intervention’s effectiveness, and more rigorous trials are now appropriate. Further evaluation of the efficiency of the HEP approach with premature infants requires more comprehensive studies such as randomized controlled trials. Additional research should also be conducted to comprehensively assess the possible effectiveness of this approach on the health of families and infants.

## Figures and Tables

**Table 1:** Description of the HEP Approach phases.

Program Phase	Description	Examples
1. Referral	Neonatologists refer infants with developmental risks.	Infants born at or before 33 weeks and six days of gestation with a corrected age between 4 and 10 months who did not exhibit any systemic disease or congenital disabilities.
2. Family Introduction	The first meeting introduces the family to the intervention approach, theory, who we are, what we do, and the HEP Approach’s general philosophy on infant development. The family’s systemic strengths and areas of limitation are identified.	Discussion with the family emphasized that every baby and family has strengths and limitations and that focusing on the family’s and baby’s strengths is critical to promote the infant’s development. For instance, when a baby performs well at socializing, the importance of using their social skills, which are their strength, is emphasized to support their motor skills. Alternatively, we emphasized how a large household is advantageous for offering various opportunities that support the infant’s development.
3. Comprehensive Assessment	A comprehensive assessment is provided in the clinic setting. Interactions between the family and the child are observed. A significant part of the assessment involves observing the child actively exploring the environment and objects with his/her hands, feet, eyes, and/or ears in different positions (supine, prone, and sitting).	The assessment process examines the child’s homeostasis, sensory processing, and emotional, motor, and cognitive development areas. Additionally, the developmental history and family interview attempt to comprehend the child’s past experiences, the services they currently receive, their future expectations, and their physical and social environment.
4. Identification of Strengths and Challenges of the Child and Parent Based on Assessment	Interpretation of the comprehensive assessment identifies the underlying child, family, and environmental factors that limit the child’s active exploration.	The infant’s strengths may include auditory or visual perception, sociability, environmental curiosity, and a responsive parent. The infant’s limitations may consist of a high arousal level, higher reactivity to movement or touch, poor body awareness, low motivation, and a worry-prone parent.
5. Formulation of Hypotheses	The hypotheses of how and why underlying factors interfere with or support the child’s development are formulated, and our hypotheses are shared with the parents.	Due to their keen awareness of vision, the infant takes great interest in everything and everyone around him. The infant’s ability to use their hands to explore novel or unfamiliar objects in strange settings was restricted due to their high level of stress.
6. Collaborative Goal-Setting	The family collaborates with the clinician to create intervention goals. Goals are based on the child’s limitations and the family’s fundamental concerns.	As the infant’s regulatory capacity develops, it will take less time for them to start exploring objects in a new environment with their hands.
7. Identification of Outcome Measures	Appropriate monitoring tools and measures are selected, and information is shared with the family.	Goal attainment scaling, Peabody Developmental Motor Scales-2, Test of Sensory Functions in Infants, etc.
8. Intervention Planning	The features of the HEP Approach are explained to the family, ensuring that they understand the program’s scientific theoretical foundation. The family receives information about their child’s HEP Approach intervention’s location, frequency, and duration.	Sessions will be 1 h per week at the clinic setting for 12 weeks, with recommendations for home activities and weekly video follow-ups.
9. Intervention	The first 1–4 sessions focus on strategies to promote homeostasis and self-regulation as well as the baby’s basic needs including sleep patterns, feeding habits, sense of safety, and regulatory capacity. Sessions 2–6 focus on organizing the physical and social environment in the home environment to support the baby’s active exploration, considering the individual differences between the infant and the family. The 4th–8th sessions guide the family to diversify the capacities the baby has acquired. They receive guidance to apply the same skill in diverse settings, experimenting with various objects and individuals. In 6–12 sessions, families are supported in making appropriate environmental arrangements, tool adaptations, object selection, division, and difficulty adjustments to support their babies’ active exploration through reflective questions provided by the clinician. The family’s self-efficacy in supporting their babies’ development is increased.	Interactions are modeled for the family to support the infant’s regulatory capacity and sense of safety. The furniture in the baby’s room is arranged to facilitate easier and safer movement, and provide objects that encourage the baby’s active exploration. Suggestions for interacting with other household members who spend time with the baby are provided. Parents can allow a baby who can sit in a laundry basket to practice sitting with the weak support of pillows at their sides, or a baby who can crawl on a flat surface, is encouraged to crawl on cushions of varying heights on the floor. Guiding questions include “which tone of voice do you think will catch your baby’s attention more and keep him/her interacting with you longer?”, or “which object do you think will motivate your baby to move more?”, or “why do you think your baby didn’t want to explore this object?”.
10. Family Home Follow-Up and Monitoring	The family is supported in incorporating these strategies into every aspect of their daily life at home. The family is monitored by sharing videos on WhatsApp. The therapist provides feedback on the family’s videos, assisting them in developing new strategies.	The parents are encouraged to remove the floor carpet after they mentioned the baby’s motions were not as strong at home as they were in the clinic when using the baby walker.
11. Evaluation of Intervention Effectiveness	At the end of the intervention period, an evaluation of the intervention’s effectiveness is completed. Outcome measures are readministered, and progress toward goals is measured.	Results might include the infant meeting 2 of 3 goals or improving their locomotion.

**Table 2:** Participant demographic information.

Variables	Mean (SD)
Enrollment corrected age (weeks)	26.06 (7.13)
Birth weight (kg)	1.48 (3.41)
Gestational age (weeks)	30.67 (2.74)
Maternal age	30.44 (2.47)
**Variables**	**N (%)**
Gender	
Male	10 (55)
Female	8 (45)
Family income	
1–2 minimum wage	3 (16.7)
3–4 minimum wage	8 (44.4)
5+ minimum wage	7 (38.9)
Mother’s educational level	
High school	4 (22.2)
Bachelor’s degree	11 (61.1)
MSc, PhD	3 (16.7)
Father’s educational level	
High school	3 (16.7)
Bachelor’s degree	15 (83.3)
MSc, PhD	0

**Table 3:** Sample goal attainment scale.

Goal	−2 (Much Less than Expected Level of Attainment)	−1 (Less than Expected	0 (Expected Level of Attainment)	+1 (Better than Expected Level of Attainment)	+2 (Much Better than Expected Level of Attainment)
1	The child takes more than 9–10 min to reach for a toy and explore it with their mother’s encouragement.	The child takes 7–8 min to reach for a toy and explore it with their mother’s encouragement.	The child takes 5–6 min to reach for a toy and explore it with their mother’s encouragement.	The child takes 3–4 min to reach for a toy and explore it with their mother’s encouragement.	The child takes 1–2 min to reach for a toy and explore with their mother’s encouragement.
2	When a new person comes into the home, the child cries immediately, and it takes more than 25 min for them to calm down.	When a new person comes into the home, the child cries immediately, and it takes 20–24 min for them to calm down.	When a new person comes into the home, the child cries immediately, and it takes 15–19 min for them to calm down.	When a new person comes into the home, the child cries immediately, and it takes 10–14 min for them to calm down.	When a new person comes into the home, the child cries immediately, and it takes less than 10 min for them to calm down.
3	Sudden sounds always (80–100%) startle the child and cause them to cry.	Sudden sounds frequently (60–79%) startle the child and cause them to cry.	Sudden sounds sometimes (30–59%) startle the child and cause them to cry.	Sudden sounds rarely (10–29%) startle the child and cause them to cry.	Sudden sounds almost never (0–9%) startle the child and cause them to cry.
4	Child maintains an independent sitting position for less than 1 min while playing with a toy.	Child maintains an independent sitting position for 1–2 min while playing with a toy.	Child maintains an independent sitting position for 3–4 min while playing with a toy.	Child maintains an independent sitting position for 5–6 min while playing with a toy.	Child maintains an independent sitting position for 7–8 or more minutes while playing with a toy.
5	Child engages in communication initiated by an adult and continues this communication cycle without distraction for less than 1 min.	Child engages in communication initiated by an adult and continues this communication cycle without distraction for 1 min.	Child engages in communication initiated by an adult and continues this communication cycle without distraction for 2 min.	Child engages in communication initiated by an adult and continues this communication cycle without distraction for 3 min.	Child engages in communication initiated by an adult and continues this communication cycle without distraction for 4 or more minutes.

**Table 4:** Outcome changes after 12 weeks of the HEP Approach intervention (*n* = 18).

	Before Intervention M (SD)	After Intervention M (SD)	*p*
PDMS-2 Scores			
Reflexes	8.17 (1.32)	10.39 (0.97)	≤0.001
Stationary	8.33 (1.32)	10.61 (1.57)	≤0.001
Locomotion	7.17 (2.25)	9.61 (1.42)	0.001
Grasping	7.72 (0.89)	10.72 (2.05)	≤0.001
Visual-Motor Integration	9.56 (1.09)	10.44 (1.33)	0.011
Gross Motor Quotient	23.78 (3.31)	30.50 (3.65)	≤0.001
Fine Motor Quotient	17.17 (1.85)	21.00 (2.72)	≤0.001
Total Motor Quotient	40.94 (4.54)	50.89 (5.21)	≤0.001
TSFI Scores			
Reactivity to Tactile Deep Pressure	8.78 (1.55)	9.94 (0.23)	0.005
Adaptive Motor Function	5.44 (2.09)	12.78 (2.15)	≤0.001
Visual Tactile Integration	6.89 (1.41)	10.11 (2.37)	≤0.001
Ocular Motor Control	1.61 (0.50)	1.94 (0.23)	0.010
Reactivity to Vestibular Stimulation	8.17 (1.97)	11.11 (1.32)	≤0.001
Total	31.00 (4.11)	45.28 (3.42)	≤0.001
AHEMD-IS Scores			
Physical Space	2.44 (1.72)	2.56 (1.85)	0.163
Variety of Stimulation	9.83 (1.29)	12.11 (1.32)	≤0.001
Fine-Motor Toys	6.56 (2.47)	8.72 (2.19)	≤0.001
Gross-Motor Toys	6.72 (2.34)	10.39 (2.47)	≤0.001
Total	25.50 (4.87)	33.83 (4.03)	≤0.001
IMMQ			
Activity	25.22 (3.38)	36.78 (3.90)	≤0.001
Exploration	14.17 (2.03)	21.22 (2.26)	≤0.001
Motivation	16.06 (1.92)	22.11 (3.14)	≤0.001
Adaptation	13.33 (1.45)	17.89 (2.02)	≤0.001
Total	68.78 (7.28)	98.00 (10.21)	≤0.001

PDMS-2: Peabody Developmental Motor Scales 2nd Edition; TSFI: Test of Sensory Functions in Infants; AHEMD-IS: Affordances in the Home Environment for Motor Development-Infant Scale; IMMQ: Infant Movement Motivation Questionnaire.

## Data Availability

All of the data are included in the manuscript.

## Supplementary Materials

The following supporting information can be downloaded at: https://www.mdpi.com/article/10.3390/jcm13185374/s1, Table S1 is the feasibility questionnaire and Table S2 is a detailed description of the components of the HEP Approach.
