# Supplementary material for: The Homeostasis-Enrichment-Plasticity (HEP®) Approach for Premature Infants with Developmental Risks: A Pre-Post Feasibility Study
Source: J Clin Med. 2024 Sep 11;13(18):5374. doi: 10.3390/jcm13185374 (PMC11432283; doi:10.3390/jcm13185374)
Supplement: Supplementary file 1 [file jcm-13-05374-s001.zip › jcm-3177260-supplementary.pdf]

Table S1-HEP Feasibility Parent Survey

HEP Approach Caregiver Feasibility and Satisfaction Survey

	**Strongly Agree**	**Agree**	**Neither Agree nor Disagree**	**Disagree**	**Strongly Disagree**
1. I was adequately informed about the intervention process and the study.					
2. I was satisfied with the HEP Approach intervention provided to my child.					
3. I would recommend the HEP Approach intervention to other caregivers of premature babies.					
4. The therapist established a good relationship with my baby.					
5. The therapist had good communication with us (caregivers).					
6. My baby was happy/having fun during therapy sessions.					
7. The HEP Approach intervention was appropriate/beneficial for my baby.					
8. Participating in the HEP Approach intervention was logistically convenient for us (time, transportation).					
9. I would like to participate in a study on the HEP Approach intervention in the future.					
10. The duration of the sessions was acceptable.					
11. It was useful for us to have the therapist follow what we did at home via video sharing.					
12. It was easy to attend in-person sessions on a weekly basis.					

13. The HEP Approach intervention approach empowered me to support my child.					
14. It was easy to embed the HEP Approach activities into daily routines.					
15. My baby was calmer following the HEP Approach intervention.					
16. The HEP Approach intervention positively affected my baby's social interaction.					
17. The HEP Approach intervention positively affected my baby's movement development.					
18. The HEP Approach intervention positively affected my baby's cognitive development (attention, problem-solving, object permanence).					
19. The HEP Approach intervention positively affected my baby's exploration skills.					
20. The HEP Approach intervention had a positive impact on our family dynamic.					
21. The HEP Approach activities were safe to use with my child.					
Do you have any suggestions for developing/improving the HEP intervention?					

Table S2-HEP Approach Intervention Guide

**Guide to Implementation of the HEP Approach Intervention**<br><br>Key Components of the HEP Approach Intervention<br>A) Parent/Caregiver coaching strategies<br>B) Activities and strategies
1. Homeostasis<br><br>The primary objective of a living body is to attain and sustain homeostasis. Homeostasis is characterized as adjusting internal environmental circumstances in response to changes in the external environment to maintain health and functionality. Homeostasis is the dynamic balance between the sympathetic and parasympathetic systems, essential for active exploration, learning, and development.<br><br>The HEP Approach prioritizes maintaining homeostasis while also considering the potential impact on all aspects of development. The homeostasis of an individual is influenced by various environmental factors, including heat, noise, and parental practices, as well as individual factors, such as health condition, sleep, eating habits, stress level, and regulating capacity. Parents are provided with guidance regarding the significance of their baby's sleep, eating, waking patterns, overall health, and regulatory abilities about the baby's active exploration, learning, and development. The therapist provides appropriate individual strategies to support homeostasis and regulatory capacities.<br><br>A. The therapist initially highlights to the family the significance of regularly assessing the overall well-being of both the infant and themselves, as well as accessing the required assistance. The therapist emphasizes the significance of developing routines in essential aspects of everyday living, such as sleep and nourishment. The therapist informs parents that their well-being directly affects the baby's self-regulating ability. Consequently, the therapist encourages parents to engage in daily activities, such as socializing with friends or walking, to enhance their self-regulation skills.<br><br>B. The HEP Approach involves the therapist providing parents with appropriate regulation strategies for the infant, such as using a pacifier or employing products that encourage the baby's independent movement and exploration, like a baby walker. Additionally, the therapist demonstrates how to use a calm attitude, comforting physical contact, rhythmic rocking, or gentle massage to regulate a disorganized baby, making the approach more practical and applicable.
2. Safety

	To effectively interact with the environment, an individual's nervous system must perceive a sense of physical and emotional safety. The presence of safety is vital in fostering the active exploration and engagement required for optimal development and learning. The therapist ensures safety by collaborating with the parents to adjust the environment according to the infant's strengths and limitations in all settings where the baby is involved. Furthermore, the therapist utilizes strategies that encourage parents to have a sense of confidence and safety. By highlighting the favorable aspects of the parent's engagement with the infant, the therapist fosters a sense of empowerment in the parent to support their infant's learning and development.<br><br>A. The therapist informs the parents about the importance of ensuring the infant's sense of security to facilitate the infant's exploration of the environment, acquisition of novel experiences, and overall sensory, motor, emotional, and cognitive development. The therapist also highlights the significance of babies relying on their parents as a source of safety, emphasizing the need for parents to feel safe themselves and be responsive to their babies.<br><br>B. Initially, the therapist encourages the parents to provide verbal or emotional support to establish a sense of safety for the baby, motivating the child to actively explore the environment when they feel unsafe. For instance, if the infant meets a new individual and feels insecure, the parent will provide information about this unfamiliar person or explain the baby's emotions to the new person while the baby looks at them. The therapist suggests adjusting the setting or equipment to facilitate the infant's active exploration. For example, a parent can enhance the baby's sense of security by placing them in a rubber tube, allowing them to sit or move comfortably between various postures. This lets the infant to freely explore different surfaces, movements, spaces, and objects.
3.	Sensory Experiences<br><br>The development and learning processes rely on extensive and varied sensory experiences. It is essential to possess sufficient sensory processing skills, which enable individuals to explore and engage with their environment. Sensory systems facilitate the transmission of precise information to the nervous system, thus enabling sensory processing and perception. They promote the individual's exploration of the environment, engagement with the context, learning, and development. Consequently, the therapist encourages the individual being worked with to actively explore, participate in, and perceive their environment. This is achieved by arranging the environment and adjusting equipment to facilitate the effective use of their sensory systems.<br><br>A. The therapist informs caregivers about the significance of various active and meaningful sensory experiences and the importance of the baby's more robust sensory systems in supporting sensory processing. The therapist highlights to caregivers the importance of the baby's strong sensory capabilities in promoting active exploration, perception, learning, and development.<br><br>B. The therapist designs environments to facilitate the baby's active exploration by utilizing their strong sensory systems. For instance, to accommodate infants with well-developed

visual perception, the therapist provides activities using upright postures like standing or sitting, selects appropriate equipment such as a basket or a baby bouncer, and demonstrates how to use them. In this way, babies can effortlessly explore their body parts, environment, and objects through their sense of sight.

4. Spatial

The physical characteristics of the space (such as the size of the home or room, support surfaces in the vertical and transverse planes, and objects and equipment in the environment) are parameters that have the potential to significantly affect active exploration, perception, learning, and development. A larger space may provide novel and various opportunities for exploration and engagement. Furthermore, space characteristics within an individual's zone of proximal development, such as the support surfaces provided by equipment, the material on the floor, or the nearby toys, provide active exploration and development opportunities.

A. The therapist informs the family about how spatial factors (such as the features, equipment, and objects in the room where the infants spent most of their time) can influence active exploration, learning, and development, as well as the importance of actively exploring various spaces.

B. To support spontaneous self-organization, stimulate perception, encourage action, and promote various active explorations, the therapist either provides spatial conditions (such as a bigger room or a room with stairs) or suggests suitable equipment (cushions of various textures, heights, and shapes to crawl on or a baby walker) tailored to the child's individual profile. The therapist facilitates development by encouraging caregivers to expand the actively explored environment.

5. Novelty

Exploration, learning, and developmental change are facilitated by novel features in the environment, such as items, toys, or surfaces, and activities that an individual can tolerate. Furthermore, encountering novel experiences offers the potential for sensory-motor, emotional, and cognitive stimulation and promotes incorporating past experiences into new situations. The therapist modifies and adapts the surroundings and tasks within the individual's zone of proximal development to encourage new opportunities for action and experiences. Moreover, the therapist collaborates with parents to provide novel experiences for their infant.

A. The therapist emphasizes to the family the importance of introducing the baby to new and varied experiences and activities. They also familiarize themselves with the baby and family's daily routines and rituals and guide them in incorporating novel aspects into these routines.

B. The therapist suggests and demonstrates strategies for promoting changes by gradually adjusting the environment and activities, which have already allowed the infant to participate in active exploration.
For example, the therapist provides and demonstrates the use of a baby walker to

engage the infant in physical activities across various areas of the house. In addition, the therapist works together with parents to modify the arrangement of furniture in order to enhance the baby's mobility and encourage their exploration in other ways. Also, as a component of the novel experience, the therapist also works with parents to include minor adjustments to the infant's daily schedule. This includes singing well-known songs with different rhythms and tones, as well as feeding the baby in different positions.

6. Challenge

Challenge is an inherent stimulant for learning and developmental change, promoting the complexity of behaviors. Experiences that are familiar, too simplistic for the child, or beyond the child's abilities do not promote effective learning. Hence, the challenge must align with the child's zone of proximal development to facilitate learning and behavioral changes. Engaging in challenging events and activities promotes the integration of all the individual's physiological systems, enhancing their learning and development ability.

A. The therapist informs parents about the importance of including environmental challenges and activities in their daily routines that are achievable for the infant and helps them determine the appropriate challenges for each situation.

B. The therapist modifies the environment and activities to align with the child's specific abilities, allowing the child to accomplish goals and succeed. For example, when an infant can sit in a laundry box, the therapist arranges and demonstrates the use of pillows to provide lateral support, enabling the child to explore new mobility opportunities. Alternatively, the therapist creates uneven floors for a crawling baby familiar with smooth surfaces to discover alternative means of movement. Additionally, if a baby can remove the covering from a parent's face, the therapist might suggest engaging in activities that uncover hidden objects.

7. Enjoyment

Learning occurs when individuals engage in meaningful, intentional, and motivating activities. Pleasurable experiences are prone to repetition, leading to multiple neurophysiological changes and adaptations, and promoting developmental advancement. Therefore, the therapist collaborates with the family to create environmental setups, activities, and adjustments in interactions that align with the child's abilities and develop favorable experiences for the child.

A. The therapist further guided the parents on organizing social and physical environments and activities to create joyful exploration experiences. For instance, the most optimal gestural response to infant behaviors was identified, and infants' favorite toys were included in the activities.

B. The importance of motivation and enjoyment in the infant’s learning and development was emphasized. The parents were guided on motivating the infant to actively explore various movement possibilities and the surrounding environment and objects. In addition, the therapist guided the parents on how to organize social and physical environments and

activities to create joyful exploration experiences. For instance, the most optimal gestural response to infant behaviors was identified, and infants' favorite toys were included in the activities.

8. Continuous Engagement

Repetition is essential for the learning process and for facilitating developmental change. Providing ample opportunities for the child to actively engage and interact with the environment regularly is crucial to promote repetition. Hence, the strategies proposed to the family should be easily implementable under various environmental and temporal circumstances. To ensure the baby's continued active exploration and participation, the therapist provides adaptations suitable for the unique characteristics of the caregivers and the environmental and temporal situations they face.

A. The therapist highlights the significance of the infant's ongoing engagement in active exploration to achieve developmental outcomes while also emphasizing the vital role of the family in facilitating this process.

B. In this context, the therapist's approach involves exploring possible opportunities that support the baby's growth, are compatible with the baby's characteristics, and fit well within the family's interactions. The goal is to improve the family's ability to think critically and solve problems by asking thoughtful questions, such as "What elements do you think influence your baby's preference for this specific toy?". As a result, the parents encourage the baby's continuous and independent exploration by providing appropriate opportunities for active exploration when alone.

9. Social

The social environment comprises all adults and children around the individual and offers significant opportunities for development and learning. Adults or children who engage in interactions that align with the child's developmental abilities within the social setting promote active exploration and engagement. It is crucial to vary the supportive social environment to facilitate learning and development. To encourage active exploration and engagement, the therapist arranges the social environment in the child's zone of proximal development to enhance the child's active exploration and engagement. The social environment consistently nurtures the child's development and learning.

A. The therapist informs the parents and close individuals around the child about the substantial influence of social interactions and relationships on learning and development. The therapist also highlights the significance of the child's broader and more varied social contextual experiences.

B. The therapist asks insightful questions to the parents to investigate the most successful ways of establishing relationships that encourage infants' active exploration and participation. For instance, a therapist could ask parents, "What specific verbal feedback do you find most effective in motivating your child to attempt new things?" or "In what ways do you provide

support to your child as they strive to achieve their goals?". These inquiries enable parents to investigate and generate strategies that promote development and learning. Parents may explore whether reducing verbal communication and increasing the use of nonverbal cues, including gestures and facial expressions, is more efficient in promoting learning. Parents may discover that their child exhibits greater curiosity and exploration when participating actively in family meetings. The therapist might demonstrate individually designed strategies to enhance parents' capacities if necessary. The therapist also encourages caregivers to provide the child with various relationship opportunities in different environments.
10. Active Engagement and Exploration<br><br>Enhanced perception and learning occur when a child actively explores the potential for taking action in the environment. The physical and social environment should encourage and facilitate the child's active exploration and engagement. The therapist creates environmental arrangements tailored to the unique characteristics of the child and the parent, enabling the child to actively explore and participate in the environment.<br><br>A. The therapist emphasizes the individuals in the baby's vicinity, environment, and available resources to promote the baby's active exploration.<br><br>B. The therapist emphasizes the importance of allocating sufficient time for active exploration to each infant so the parents can investigate their child's unique and necessary time to engage. The therapist guides parents to find appropriate environments, equipment, tools, communication methods, and natural rewards to encourage the infant's active exploration. For instance, one parent may propose relocating all the carpets to facilitate the infant's mobility with the baby walker. Another parent discovers that the infant demonstrates better object exploration abilities while sitting than lying on their back.
